# Causality, Severity, and Avoidability of Adverse Drug Reactions in Hospitalized Children: A Prospective Cohort Study

**DOI:** 10.7759/cureus.33369

**Published:** 2023-01-04

**Authors:** Saurabh Gupta, Syed A Zaki, Sanjeevani Masavkar, Preeti Shanbag

**Affiliations:** 1 Pediatrics, Lokmanya Tilak Municipal Medical College and General Hospital, Mumbai, IND; 2 Pediatrics, All India Institute of Medical Sciences, Bibinagar, Hyderabad, IND

**Keywords:** pharmacovigilance, monitoring, prospective cohort study, incidence, children, adverse drug reactions

## Abstract

Background: Adverse drug reactions are an important cause of morbidity and mortality in all patients. Information regarding adverse drug reactions in the pediatric age group, especially with regard to the drugs involved and the clinical presentations is scanty. The aim of our study is to determine the incidence of adverse drug reactions and to study their features in terms of causality, type, severity, avoidability, drugs implicated and their clinical presentations.

Methods: The study was carried out on patients admitted to the pediatric ward and the pediatric intensive care unit over a one-year period (January 1, 2013 to December 31, 2013). Patients either presenting with or developing an adverse drug reaction in the hospital were included in the study.

Results: The incidence rate for adverse drug reaction causing hospital admission was 1.79% (95% CI 1.48, 2.16) whereas it was 1.23% (95% CI 0.97, 1.53) for children exposed to a drug during their hospital stay. Type B (bizarre or idiosyncratic type) was seen in 114 (62.6%) of the ADRs whereas 53 (29.1%) were of type A (augmented pharmacologic effect). Severe ADRs were seen in 25 (13.7%) of the total ADRs. ADR was responsible for the death of two patients. 15.4% were rated as avoidable. Anti-microbials were the most common group responsible for ADRs (43.4%), followed by drugs acting on the immune system (15.9%) and drugs acting on the nervous system (14.3%). The most common ADRs were metabolic (29.3%) followed by neurological (17.6%).

Conclusions: Adverse drug reactions can occur in a substantial proportion of hospitalized patients with some of them being severe and potentially avoidable. Awareness among physicians should be encouraged regarding monitoring, documentation and notification of adverse drug reactions.

## Introduction

“Primum Non Nocere” (First, do no harm.) is a fundamental principle throughout the world for healthcare professionals. Every drug which the physician uses is double-edged, and every cure has potential harm. Every drug has the potential to have an adverse effect, for which awareness and monitoring are necessary. Adverse drug reactions (ADRs) cause significant morbidity and mortality in patients of all ages. Before new drugs are launched in the market, clinical trials (postauthorization safety studies) for ADRs are conducted primarily in adults. ADRs in children may differ from those in adults due to age-dependent pharmacokinetics and pharmacodynamics of drugs. Further, children are often prescribed medications that are off-label putting them at a greater risk potentially for ADRs [1-3]. The majority of the time, post-marketing surveillance remains the predominant way to detect ADRs specific to children. Spontaneous reporting systems for ADRs are subject to under-reporting even in the case of severe ADRs [4]. Although the national pharmacovigilance system was introduced in India in 2010, significant under-reporting occurs here as well [5,6]. The spectrum of disease in children in each country is not exactly similar. For example, in India, the spectrum of diseases is different from that in developed countries, with infectious diseases predominating. Hence, it is important to identify ADRs to drugs used in the treatment of diseases endemic to this country. Though children constitute 40% of the population of India, information regarding ADRs, the drugs involved, and the clinical presentations in this age group, are scanty [2-4]. ADRs can result in unplanned hospital outpatient visits, unplanned hospital admission, and economic losses to the health service system [2]. Hence, an improved understanding of the severity and causality of ADRs is crucial. Studies monitoring ADRs in children can facilitate improved strategies for better management of ADRs. Additionally, such studies may improve safe and effective treatment practices in the pediatric population.

The aim of this study was to prospectively identify ADRs in hospitalized children during a one-year period, to determine the incidence of ADRs, and to characterize their features in terms of causality, type, severity, avoidability, drugs implicated, and clinical presentations.

## Materials and methods

This prospective study was carried out over a one-year period (January 1, 2013 to December 31, 2013) at Lokmanya Tilak Municipal Medical College and General Hospital, a tertiary care teaching hospital in Mumbai, India. The study was approved by the institutional research ethics committee (reference No. SRS/17/12). We prospectively screened all admissions to the pediatric ward and pediatric intensive care unit (PICU). The pediatric ward and PICU admit 6,000-7,000 patients (one month to 12 years) per year. Neonates and pediatric surgical patients are admitted elsewhere in the hospital and are managed by different departments. The case records, drug charts, and laboratory data of all admissions in the previous 24 hours were reviewed daily for ADRs, by one of the authors. In addition to the screening of the author, our institute also has a pharmacovigilance department that routinely monitors ADRs in the hospital. This double-check ensured that the chance of missing any case of ADR was minimal. The records of patients who were subsequently reported to have ADRs were also reviewed in a similar manner. ADRs were defined using the Edwards and Aronson definition which is “an appreciably harmful or unpleasant reaction resulting from an intervention related to the use of a medicinal product, which predicts hazard from future administration and warrants prevention or specific treatment, or alteration of the dosage regimen or withdrawal of the product” [7]. We excluded patients admitted with intentional or accidental poisoning, medication errors, and drug abuse. We recorded the following details of each patient: demographic data, drug history, and clinical details, and relevant information about the suspected reaction, its onset, dose, duration, and temporal association with drug intake.

We assessed causality using the World Health Organization-Uppsala Monitoring Centre (WHO-UMC) system for standardized case causality assessment [8]. We determined the type of ADR using the Rawlins and Thompson classification and classified the severity of ADRs as per the modified Hartwig and Siegel severity scale [9,10]. Severity levels 1 and 2 were classified as mild, levels 3 and 4 were classified as moderate, and levels 5-7 were classified as severe. We assessed avoidability as per the scale described by Hallas et al. [11]. We reported the outcome, i.e., causality, type of reaction, severity, and avoidability by consensus among the three assessors. Our estimate for the overall incidence was based on the sum of probable and definite ADRs. Incidence was calculated by dividing the number of admissions in which at least one ADR occurred, by the total number of admissions in the same year, regardless of drug exposure.

Patients were included after taking informed consent from the parent or guardian and oral assent from children between seven and twelve years of age. All ADRs were reported to the ADR Monitoring Centre of the hospital (established under the Pharmacovigilance Programme of India). Descriptive statistics were used to describe the data.

## Results

The pediatric ward and pediatric intensive care unit (PICU) admitted 6026 patients during the study period. Of these, 182 admissions experienced an ADR giving an overall incidence of 3.02%. ADRs occurred before admission in 108 of 182 (59.3%) admissions, whereas in 74 admissions (40.7%), the ADRs occurred after hospitalization. Thus, the incidence rate for ADRs causing hospital admission was 1.79% (95% CI 1.48, 2.16) whereas it was 1.23% (95% CI 0.97, 1.53) for children exposed to a drug during their hospital stay. Tables 1, 2 show the age and sex of hospitalized children with ADR. There was no age or gender preponderance. Out of 182 cases who developed ADR, 27 cases were direct admissions to PICU. Forty-four cases who developed ADR in the ward were transferred to PICU, either due to the severity of ADR or due to their underlying co-morbid condition.

**Table 1:** Age distribution of hospitalized infants and children with adverse drug reaction

Age	Total number of children admitted	Number with ADR (%)
1 month-1 year	1495	45 (3.0)
1-5 years	2178	64 (2.9)
>5 years	2353	73 (3.1)
Total	6026	182 (3.02)

**Table 2:** Sex distribution of hospitalized infants and children with adverse drug reaction

Age	Number of children with ADR	Male (%)	Female (%)
1 month-1 year	45	23 (51)	22 (49)
1-5 years	64	34 (53)	30 (47)
>5 years	73	38 (52)	35 (48)
Total	182	95 (52)	87 (48)

Table 3 shows the causality, type of reaction, severity of ADRs and avoidability in our patients. Bizarre or idiosyncratic types of reactions were reported in 114 of 182 (62.6%) patients. Reactions classified as levels 5 to 7 were considered severe. In our study, 25 of 182 reactions (13.7%) were severe, with two ADRs resulting in death. Both these ADRs were classified as being unavoidable, severe type, and having possible causality. One death was in a seven-year-old girl child who developed toxic epidermal necrolysis following the administration of ciprofloxacillin. The other death was in a nine-year-old boy with tetanus, who developed severe bradycardia and cardiac arrest following the use of vecuronium for paralysis during ventilation. In our study, 28 of 182 (15.4%) of recorded ADRs were rated as avoidable. In 12 patients, the ADRs were rated as definitely avoidable. Three children received amoxicillin (two) and amoxicillin-clavulanic acid, where signs and symptoms were typical of a viral illness. In three patients, vancomycin was started as empiric therapy for meningitis despite there being no clinical evidence of staphylococcal disease. In two non-oncological patients with vomiting, metoclopramide was used as first-line therapy and resulted in dystonia. Ibuprofen was used for fever in a dehydrated patient with fever and vomiting and resulted in acute renal failure. Convulsions occurred in a patient with acute asthma, who received a loading dose of aminophylline, despite the child having been on oral aminophylline prior to admission. We found that one child who received intravenous calcium gluconate had extravasation of the drug causing local inflammation. Also, one patient who received intravenous midazolam for convulsions had respiratory depression due to the rapid administration of the drug. Sixteen ADRs were deemed to be possibly avoidable. Ten patients who received home nebulization with salbutamol developed tremors which could have been prevented by adequate counselling. Nine patients on furosemide developed hypokalaemia which could have been prevented by potassium supplementation. The total avoidable (possibly and definitely avoidable) group had 28 cases. Out of these 19 cases (67%) ADRs were of a mild type, and nine cases were moderate type. The type of reaction was type A in 13 cases and type B in 15 cases. On causality assessment, eight cases of ADR were classified as “certain,” 15 cases were classified as “possible,” and three cases were classified as “probable.”

**Table 3:** Causality assessment, type of reaction, severity and avoidability of adverse drug reaction

Causality assessment (WHO-UMC system of causality assessment)	n (%)
Certain	43 (21.4 )
Probable	139 (69.2 )
Possible	14 (7.0 )
Unlikely	5 (2.4 )
Unclassifiable	0 ( 0.0)
Type of reaction (n=182)	
Type A - Augmented pharmacologic effect	53 ( 29.1)
Type B - Bizarre effect (idiosyncratic effect)	114 ( 62.6)
Type C - Chronic effect	15 ( 8.2)
Type D - Delayed effect	0 ( 0)
Type E - End of treatment effect	0 (0 )
Severity of ADR (n=182)	
Mild (Levels1 and 2)	23 (12.6 )
Moderate (Levels 3 and 4)	134 (73.6 )
Severe (Levels 5-7)	25 (13.7 )
Avoidability (n=182)	
Not avoidable	154 (84.6 )
Definitely avoidable	12 (6.6 )
Possibly avoidable	16 ( 8.8)

Anti-infectives were the most common group responsible for ADRs in 79 admissions (43%), with antibiotics being the most common (34 of 79), i.e., 43%. Immunomodulators were responsible for ADRs in 29 admissions (16%). Drugs acting on nervous system were responsible for ADRs in 26 admissions (14%) with antiepileptics being the most common (18 of 26), i.e., 69%. ADRs associated with bronchodilators, diuretics, and NSAIDs were seen in 11, nine and eight admissions, respectively. The most common ADRs were metabolic followed by neurological. Table 4 lists the different organs affected by ADRs.

**Table 4:** System-wise distribution of adverse drug reaction

No.	System	n (%)
1	Metabolic	55 (29.26)
2	Central and peripheral nervous system	33 (17.55)
3	Skin and appendages	32 (17.02)
4	Gastro-intestinal, liver and biliary tract	32 (17.02)
5	Haematologic	25 (13.29)
6	Cardiovascular/Haemodynamic	11 (5.85)

 Tables 5-7 list the various drugs and the associated ADRs.

**Table 5:** Adverse drug reactions associated with antibacterial drugs

No	Drug	n (%)	Type of adverse drug reaction
1	Ceftriaxone	8 (4.3)	Anaphylaxis
			Biliary pseudolithiasis
			Rash
			Rigors
2	Amoxicillin-clavulinic acid	6 (3.2)	Diarrhoea
			Rash
			Angio-neurotic oedema
3	Vancomycin	5 (2.7)	Red man syndrome
4	Piperacillin-tazobactum	3 (1.6)	Thrombocytopenia
			Hypokalaemia and metabolic alkalosis
5	Meropenem	3 (1.6)	Hypokalaemia and metabolic alkalosis
6	Co-trimoxazole	2 (1%)	Hypoglycaemia and convulsions
			Megaloblastic anaemia
7	Chroramphenicol	2 (1%)	Pancytopenia
8	Amoxicillin	1 (0.5)	Rash
9	Cefotaxime	1 (0.5)	Rash
10	Cephalexin	1 (0.5)	Rash
11	Ciprofloxacin	1 (0.5)	Toxic epidermal necrolysis and death
12	Amikacin	1 (0.5)	Acute renal failure

**Table 6:** Adverse drug reactions associated with antimalarials, antifungals and antiretroviral drugs

No	Drug	n (%)	Type of adverse drug reaction
I	Antimalarials		
Ia	Chloroquine phosphate	8 (4.3)	Epigastric pain and vomiting
			Rash
			Dystonia
Ib	Clindamycin	4 (2.1)	Rash
			Anaphylaxis
			Hyponatraemia
Ic	Artesunate	2 (1%)	Hyponatraemia due to natriuresis
II	Antifungals		
IIa	Amphotericin B	9 (5)	Acute renal failure (elevated serum creatinine)
			Hypokalaemia
			Prolonged PR interval
III	Antiretrovirals		
IIIa	Zidovudine	4 (2.1)	Anaemia
IIIb	Stavudine	2 (1%)	Pancreatitis
			Lactic acidosis
IIIc	Lamivudine	1 (0.5)	Pancreatitis

**Table 7:** Adverse drug reactions associated with drugs acting on the nervous system

No	Drug	n (%)	Type of adverse drug reaction
I	Antiepileptics		
Ia	Carbamazepine	6 (3.2)	Drowsiness
			Hypocalcaemia
			Dystonia
			Ataxia
			Weight gain
Ib	Phenytoin sodium	5 (2.7)	Ataxia
			Nystagmus
			Gum hyperplasia
			Rash
Ic	Phenobarbitone	4 (2.1)	Ataxia
			Nystagmus
			Erythema multiforme
			Hypotension
Id	Sodium valproate	3 (1.6)	Hepatitis
II	Anaesthetic agents		
IIa	Ketamine	3 (1.6)	Fasciculations of tongue
			Vomiting
			Agitation during recovery
III	Membrane stabilisers		
IIIa	Intravenous magnesium sulphate	2 (1%)	Hypercalciuria and hypocalcaemia
IV	Muscle relaxants		
IVa	Vecuronium	1 (0.5)	Severe bradycardia and cardiac arrest
IVb	Baclofen	1 (0.5)	Acute flaccid paralysis

## Discussion

This prospective observational study is one of the largest in children in India to evaluate causality, type of reaction, severity, avoidability, type of drug, and clinical presentation. In our study, the overall incidence of ADRs was 3.02%. The incidence rate for ADRs causing hospital admission was 1.79% (95% CI 1.48, 2.16), whereas it was 1.23% (95% CI 0.97, 1.53) for children exposed to a drug during their hospital stay. Smyth et al. in a systematic review of ADRs in children gave a pooled estimate of the incidence rate for ADRs causing hospital admission to be 2.9% (95% CI 2.6, 3.1). For ADRs occurring in hospitals, they found incidence rates ranging from 0.6% to 16.8% of patients [12]. Similar to other studies, we found no age or sex predisposition for ADRs. A majority, i.e., 114 of 182 (62.6%) of the ADRs were of the type B (bizarre or idiosyncratic type) whereas 53 of 182 (29.1%) were of type A (augmented pharmacologic effect). Type B ADRs were most common with anti-infectives (44) followed by drugs acting on the nervous system (23). Gallagher et al., however, found 238/249 (95.6%) of ADRs to be of type A and 11/249 (4.4%) to be of type B. Ninety-four of the type A ADRs were of definite causality with 80 of these 94 being related to oncology drugs [13]. Twenty-three of our 182 ADRs were of the mild variety, 41 were moderate and 25 were severe. In contrast, Kurian et al. reporting from Mysore, India, found mild ADRs in 82.5%, moderate in 17.18% and severe in none [14]. In our patients, mild ADRs were observed in 23 patients, all of whom developed the reaction after exposure to a drug in the hospital. It is possible that those who presented with mild ADRs to the hospital were dealt with on an outpatient basis. Gallagher et al. found 223 of 249 patients to have ADRs of level 3 severity, 14 with level 4, three with level 5, and none with level 6 [13].

In 28 of 182 patients, the ADRs were deemed avoidable with 12 being definitely avoidable. Analysis of these ADRs in our study emphasizes the importance of following rational prescribing guidelines, improving monitoring for ADRs, increasing awareness about potential reactions of drugs, and spending time on patient education. The majority of the ADRs seen in our study were due to anti-infectives (43.4%) followed by drugs acting on the immune system (15.9%) and drugs acting on the nervous system (14.3%). Smyth et al. in their systematic review of ADRs in children found the proportions of ADRs due to anti-infectives ranging from 3.5%-66.6% for “causing admission” studies (17 studies) and 8.6%-100% for “in hospital” studies (24 studies) [12]. Gallagher et al. from the UK found oncology-related drugs to be the most common cause of ADRs (44.2%) [13]. The wide distribution of drug classes responsible for ADRs in children in various settings probably reflects the type of admissions and the prescribing practices in the institution.

In our study, the most commonly observed ADRs were metabolic disturbances, followed by central and peripheral nervous system manifestations. Other authors have described gastrointestinal manifestations to be the most commonly observed ADRs, followed by dermatologic manifestations [14-16]. The most common metabolic disturbance seen was hypokalemia. In nine patients, this was due to furosemide. Eleven patients on anti-infectives had hypokalemia, seven of them due to amphotericin B. Meropenem was responsible in three patients and piperacillin-tazobactam in one patient. Patients on diuretics and amphotericin are routinely monitored for hypokalemia. However, pediatricians should be aware of this ADR with the β lactam group of antibiotics since chronic hypokalemia may cause electrocardiographic changes and/or weaning failure in the pediatric intensive care unit. The β lactam group of antibiotics are non-absorbable anions that generate an increased trans-tubular potential difference at the distal tubule causing an increase not only in potassium secretion but also hydrogen ion secretion resulting in metabolic alkalosis and hypokalemia [17]. Meropenem causes metabolic alkalosis by the same mechanism due to structural similarities [18]. Some rare ADRs were also seen in the course of this study. Common ADRs seen with chloroquine include gastrointestinal problems, headache, blurring of vision, and itching [19]. Dystonia was observed in a patient with confirmed uncomplicated vivax malaria after a standard dose of oral chloroquine phosphate. Singhi et al. have also described an extrapyramidal syndrome following a standard dose of chloroquine [20]. Dystonia was also seen in a developmentally normal patient with epilepsy on therapeutic doses of carbamazepine. Dystonic reactions with therapeutic doses of carbamazepine have been described by other authors as well [21,22]. Secondary dystonia is most commonly due to drugs with anti-dopaminergic properties such as first and second-generation antipsychotics. Less frequently implicated are other drug classes like anti-emetics such as metoclopramide. Co-trimoxazole (trimethoprim-sulfamethoxazole), a commonly prescribed antimicrobial is well tolerated in most patients. However, serious adverse events related to its use have been described. Hypoglycemia is a rare but potentially life-threatening complication of therapy [23]. We report a patient of AIDS, with prolonged hypoglycemia and seizures associated with co-trimoxazole, used for the treatment of Pneumocystis jirovecii pneumonia. Serum creatinine in our patient was normal. In previously reported cases of co-trimoxazole-induced hypoglycemia, renal insufficiency was the most prevalent predisposing risk factor (93%). Serum insulin levels were elevated or inappropriately normal in 88% of the patients in which they were measured, suggesting a sulfonylurea-like effect of co-trimoxazole as the mechanism of hypoglycemia [23]. Dosage adjustments should be made when prescribing co-trimoxazole to patients with renal dysfunction.

The strength of this study is that we have prospectively investigated ADRs in children using active search methods. This method facilitated the daily monitoring of patients by trained physicians. Also, such prospective studies allow greater detection of ADR as compared to retrospective studies. Our study has certain limitations. We have excluded neonates and pediatric surgical patients since they are admitted elsewhere in the hospital and managed by different departments. Studies have also shown that genetic factors play a role in determining individual susceptibility to both dose-dependent and dose-independent ADRs. Some of these determinants include kinetic factors (gene polymorphisms in cytochrome P450 enzymes), and dynamic factors (polymorphisms in drug targets) [24]. We have not assessed these factors due to financial constraints. Some of the drug-ADR combinations listed in Tables 5, 6 have low numbers thereby mandating further large-scale pharmacovigilance studies in children. Currently, we do not have an electronic health record system in our institution. Although this system can measure incidence rates of ADEs, it is not adequate to detect preventable ADRs before patient harm occurs. Newer methods involving computer monitors and electronic triggers can enable researchers to catch preventable ADRs and take corrective action [25].

## Conclusions

In conclusion, we have shown that ADRs occur in a small but substantial proportion of hospitalized children. Some of these ADRs are serious and potentially avoidable. More attention needs to be given to good prescribing practices to prevent avoidable ADRs. Active search while taking clinical ward rounds with a focus on specific drugs associated with ADR can increase the rates of detected reactions. Electronic health record systems as sources of data for ADR detection. can measure incidence rates of ADRs but is not adequate to detect preventable ADRs before patient harm occurs. Finally, awareness amongst physicians should be encouraged regarding monitoring, documentation and notification of ADRs.
